# Identification of Senescent Cells in Peri-Implantitis and Prevention of Mini-Implant Loss Using Senolytics

**DOI:** 10.3390/ijms24032507

**Published:** 2023-01-28

**Authors:** Niuxin Yang, Masato Nakagawa, Aki Nishiura, Masahiro Yamada, Hidetoshi Morikuni, Yoshitomo Honda, Naoyuki Matsumoto

**Affiliations:** 1 Department of Orthodontics, Osaka Dental University, 8-1 Kuzuhahanazonocho, Hirakata 573-1121, Japan; 2 Department of Oral Anatomy, Osaka Dental University, 8-1 Kuzuhahanazonocho, Hirakata 573-1121, Japan; 3 Division of Molecular and Regenerative Prosthodontics, Tohoku University Graduate School of Dentistry, 4-1 Seiryomachi, Sendai 980-8575, Japan

**Keywords:** orthodontics, mini-implant, peri-implantitis, senescence, senolytics, Dasatinib, Quersetin

## Abstract

Peri-implantitis is a disease that causes the detachment of orthodontic mini-implants. Recently, stress-induced senescent cells have been reported to be involved in various inflammatory diseases. Senescent cell-eliminating drugs, termed “senolytics”, can improve the symptoms of such diseases. However, the relationship between peri-implantitis and senescent cells remains unclear. In this study, we evaluated the presence of senescent cells in a rat peri-implantitis model developed with a gum ring. The effect on bone resorption and implant loss was also investigated with and without senolytics (Dasatinib and Quercetin). The number of senescence markers (p19, p21, and p16) was found to increase, and implant detachment occurred in 24 days. After the administration of senolytics, the number of senescence markers decreased and implant detachment was inhibited. This study suggests that senescent cells aggravate peri-implantitis and senolytic administration latently reduces implant loss by inhibiting senescence-related mechanisms.

## 1. Introduction

Ensuring a fixed source is one of the central elements of biomechanics in dynamic orthodontic treatment [1]. Orthodontic mini-implants only fixation source that allow absolute fixation are an essential to current orthodontic treatment [2,3,4]. However, there are still many failures, mainly due to mobility and detachment caused by the progression of peri-implantitis [5].

Peri-implantitis—a disease characterized by inflammation [6] and alveolar bone loss—is usually caused by poor plaque control [5,7]. In peri-implantitis, chronic bacterial infections—such as *P. gingivalis* infection—cause bone resorption via RANKL through a complex signaling network [8]. As with other chronic inflammatory diseases, peri-implantitis progression is due to inflammatory cytokines such as IL-1β, TNF-α, and IL-6 [9]. Thus, peri-implantitis research has focused on bacterial infection, inflammatory cytokines, and osteoclasts.

Recently, the relationship between various chronic inflammatory diseases and cellular senescence has been under the spotlight. Several studies have shown that cells that are exposed to dangerous stresses that cause DNA damage or cell cycle arrest induce cellular senescence [10,11,12]. A phenomenon called senescence-associated secretory phenotypes (SASPs) has been reported in which senescent cells secrete various factors, including inflammatory cytokines, chemokines, growth factors, and matrix metalloproteinase [13]. Proinflammatory cytokines in SASPs, such as TNF-α and IL-6, cause chronic inflammatory diseases such as atherosclerosis, rheumatoid arthritis, type 2 diabetes, osteoporosis, and Alzheimer’s disease [14,15,16,17,18,19]. With elucidation of the relationship between senescence and diseases, senescent cell-eliminating drugs, termed “senolytics”, have been developed and recently investigated to treat various chronic diseases [20,21]. The combination of Dasatinib and Quercetin (D + Q), the first senolytic discovered [22], has attracted significant attention as a clinically applicable drug to control senescence [23,24].

However, the relationship between peri-implantitis and cellular senescence remains to be elucidated. Thus, this study aimed to evaluate the appearance of senescent cells in a rat peri-implantitis model developed using a gum ring (GR). Furthermore, we investigated whether the administration of senolytics could improve the mobility and detachment caused by peri-implantitis.

## 2. Results

### 2.1. Plaque Staining

**Figure 1:**
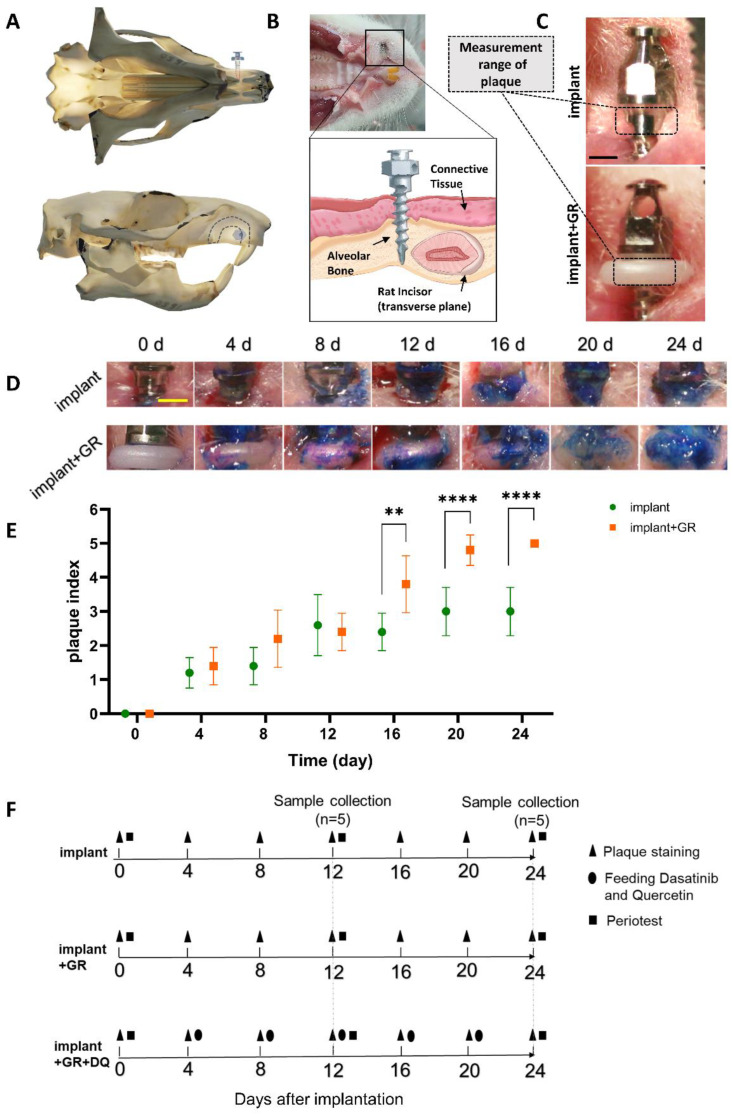
Establishment of peri-implantitis model. (**A**) Three-dimensional schematic illustration of maxillary buccal implant site in a rat. (**B**) Representative photograph and schematic illustration of the implant site. (**C**) Measurement range of plaque index. Scale bar: 1 mm. (**D**,**E**) Representative photograph and quantitative graph of plaque index analysis. Scale bar: 1 mm. Data are presented as mean with standard deviation (SD), (n = 5). Two-way analysis of variance (ANOVA) with Sidak’s multiple comparisons test; ** *p* < 0.01, **** *p* < 0.0001. (**F**) Schematic diagram of the experimental procedure.

To quantitatively analyze plaque adhesion around the implant, plaque was stained and measured at the defined area around the GR every 4 days (Figure 1A–F). Criteria based on previous reports [25] were used for the plaque measurement (Table 1). Plaque increased over time in both the implant and implant + GR groups, with a predominant increase in the implant + GR group after day 16. This result indicates that the placement of the GR between the implant and oral mucosa increased the adhesion of the plaque around the implant.

### 2.2. Inflammatory Cell Infiltration and Bone Resorption

#### 2.2.1. Hematoxylin–Eosin (HE) Staining

**Figure 2:**
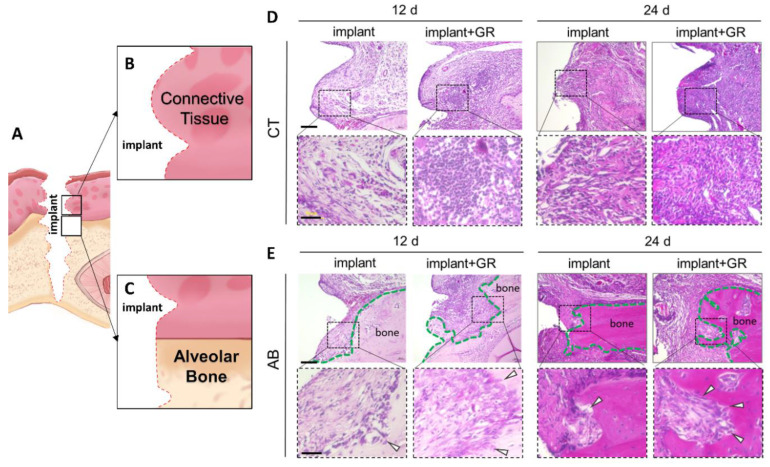
Hematoxylin–eosin staining (HE) evaluation of peri-implant tissue. (**A**) Schematic illustration of detached implant site. (**B**,**C**) Schematic illustration of peri-implant connective tissue (CT) region and region near the alveolar bone (AB). (**D**,**E**) Representative HE images of the CT and AB regions. Lower: high magnification. Scale bar: 100 μm (Upper), 50 μm (Lower). Green dotted lines: alveolar bone. Arrow heads: bone resorption-like lacunae.

For subsequent histological analysis, the peri-implant tissue was divided into two regions of interest: the region of subepithelial connective tissue (CT) and the region near the alveolar bone (AB) (Figure 2A–C). Using HE staining, we analyzed the inflammatory cellular infiltrate of implants with increased plaque attachment due to the placement of GR. Inflammatory cell infiltrates were observed in the CT and AB areas in both the implant and GR groups, indicating that chronic inflammation occurred as in clinical peri-implantitis (Figure 2D). In addition, an irregular resorption-like lacunae (arrow) was observed at the margins of the AB (Figure 2E).

#### 2.2.2. Inflammatory Markers by Immunohistochemistry Staining

**Figure 3:**
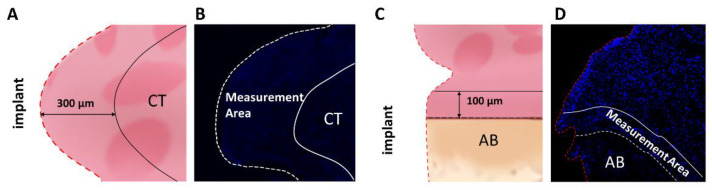
Detailed measurement area of quantitative histological analysis. (**A**–**D**) Schematic illustration and representative immunofluorescent images of the measurement area of the peri-implant CT region and region near AB.

**Figure 4:**
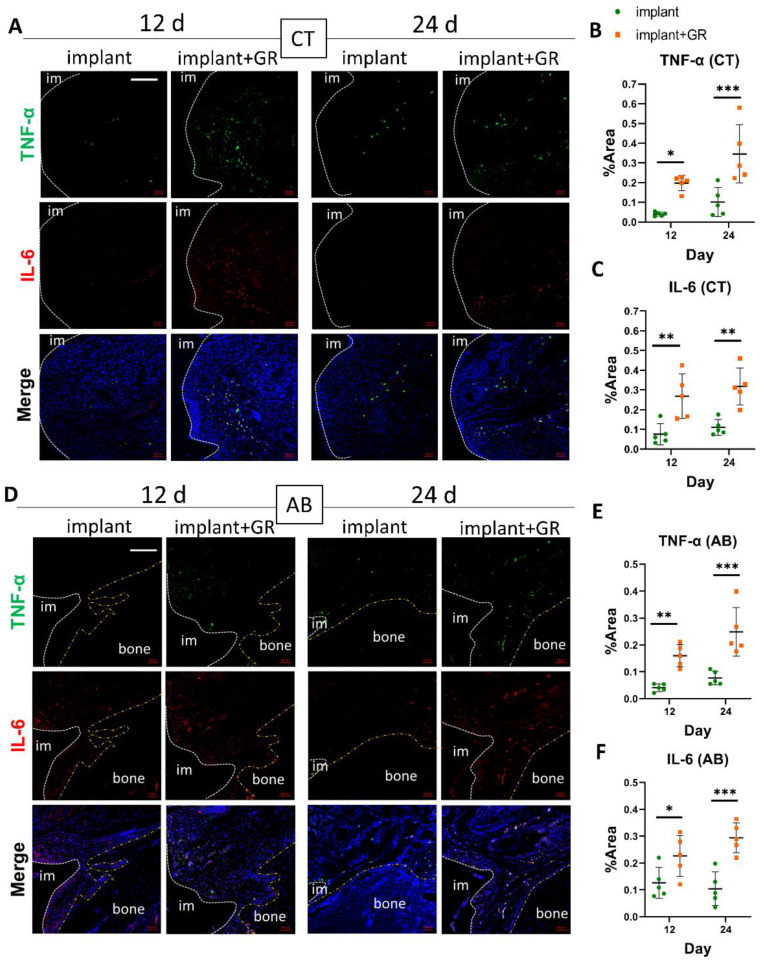
Validation of inflammatory cytokines by immunofluorescence. (**A**,**D**) Representative immunofluorescent images of the CT and AB regions against inflammatory markers TNF-α, IL-6, and 4′,6-diamidino-2-phenylindole (DAPI) for nuclear. im: implant; bone: alveolar bone. Scale bar: 100 μm. (**B**,**C**,**E**,**F**) Quantitative analysis of TNF-α- and IL-6-positive areas in the CT and AB regions. Data are presented as mean with SD Two-way ANOVA with Sidak’s multiple comparisons test; * *p* < 0.05, ** *p* < 0.01, *** *p* < 0.001.

Fluorescent immunohistochemistry (IHC) for IL-6 and TNF-α was performed to confirm the location of inflammatory cytokines in the inflammatory cell infiltrates. For the quantitative analysis of IHC-positive cells, the same areas of CT (300-μm perimeter) and AB (100-μm perimeter) (Figure 3) were measured. The range of IL-6-positive cells was significantly wider in the GR group than in the implant group. The extent of IL-6-positive cells was not significantly different between the 12- and 24-day groups for both CT and or AB (Tables S1 and S3). Compared to the implant group, the GR group showed a predominant positive-area increase at 12 and 24 days (Figure 4). These results indicate that the placement of the GR between the implant and oral mucosa enabled the development of a peri-implantitis model with chronic inflammation and bone resorption around the implant.

### 2.3. Identification of Senescent Cells by IHC Staining

**Figure 5:**
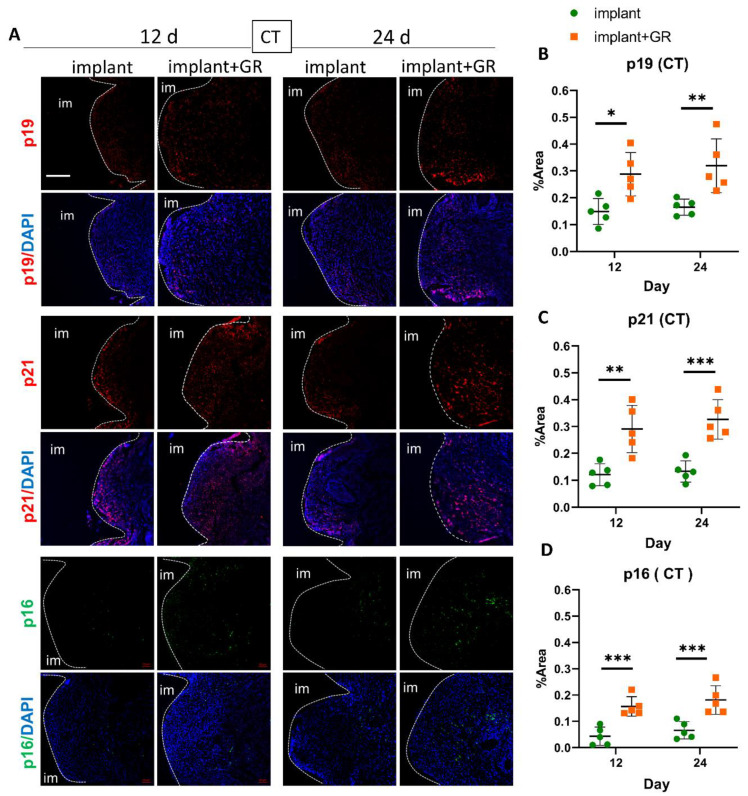
Identification of cellular senescence in CT by immunofluorescence. (**A**) Representative immunofluorescent images of the CT region against senescent markers p19, p21, p16, and DAPI. im: implant. Scale bar: 100 μm. (**B**–**D**) Quantitative analysis of p19-, p21-, and p16-positive areas in the CT region. Data are presented as mean with SD Two-way ANOVA with Sidak’s multiple comparisons test; * *p* < 0.05, ** *p* < 0.01, *** *p* < 0.001.

**Figure 6:**
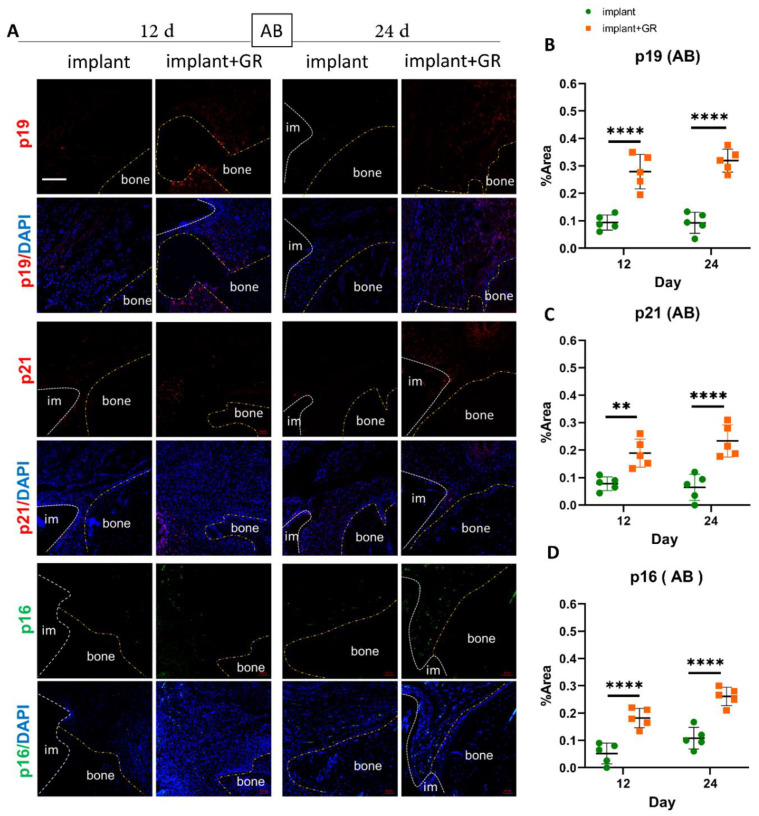
Identification of cellular senescence in AB by immunostaining. (**A**) Representative immunofluorescent images of the AB region against senescent markers p19, p21, p16, and DAPI. im: implant; bone: alveolar bone. Scale bar: 100 μm. (**B**–**D**) Quantitative analysis of p19-, p21-, and p16-positive areas in the AB region. Data are presented as mean with SD Two-way ANOVA with Sidak’s multiple comparisons test; ** *p* < 0.01, **** *p* < 0.0001.

IHC staining for senescent markers p19, p21, and p16 was performed to analyze the presence of senescent cells in peri-implantitis (Figure 5 and Figure 6). In the CT (Figure 5) and AB (Figure 6) regions, p19-, p21-, and p16-positive cells increased in the GR group compared to the implant group (Tables S1 and S3). In the GR group, there were no statistical differences between the p19-, p21-, and p16-positive areas at 12 days, whereas the mean value increased (Tables S1 and S3). These results indicate that the peri-implantitis model with the placement of GR showed an increase in senescent cells.

### 2.4. Effectiveness of Senolytics for Peri-Implantitis

#### 2.4.1. Inflammatory Markers by IHC Staining after Dasatinib + Quercetin (D + Q) Administration

**Figure 7:**
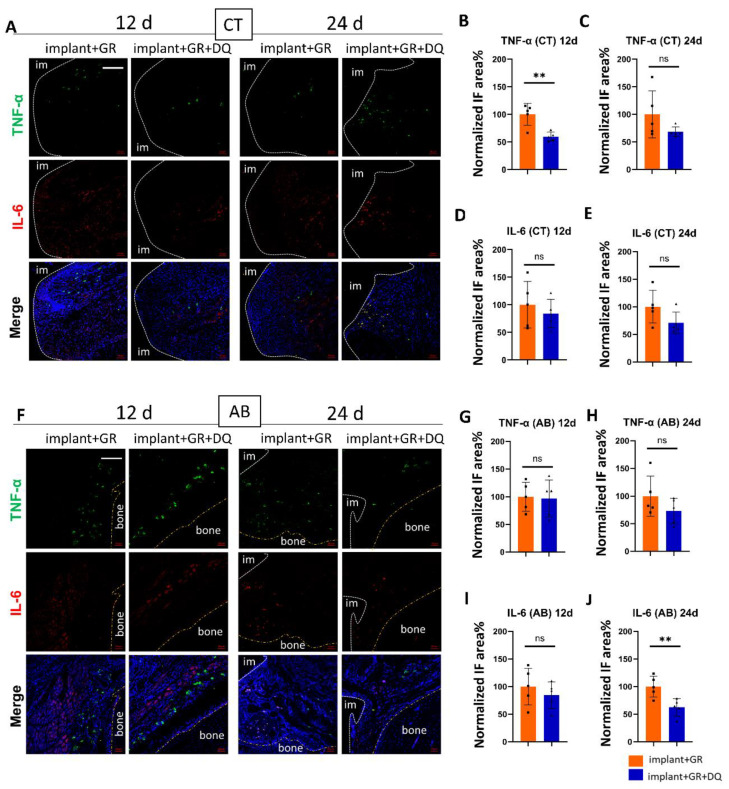
Verification of inflammatory cytokines after D + Q administration. (**A**,**F**) Representative immunofluorescent images of the CT and AB regions against inflammatory markers TNF-α, IL-6, and DAPI. im: implant; bone: alveolar bone. Scale bar: 100 μm. (**B**–**E**,**G**–**J**) Quantitative analysis of TNF-α- and IL-6-positive areas in the CT and AB regions on day 12 (12 d) and day 24 (24 d) after implantation. IF: immunofluorescence. Data are presented as mean with SD Student’s *t*-test; ** *p* < 0.01, ns: no significance. Square and triangle plots are individual value of each group.

The GR groups and GR with D + Q groups were compared to examine the effect of senolytics on inflammatory cytokines in peri-implantitis. In CT, the positive area in TNF-α on day 12 decreased after the administration of D + Q (Figure 7A–E). In AB, IL-6 staining showed a significant decrease in the area of positive staining in the D + Q group compared to the GR group on day 24 (Tables S1 and S3). There were no statistical differences between the other groups. However, all groups showed a decrease in mean values (Figure 7F–J).

#### 2.4.2. Identification of Senescent Cells by IHC Staining after D + Q Administration

**Figure 8:**
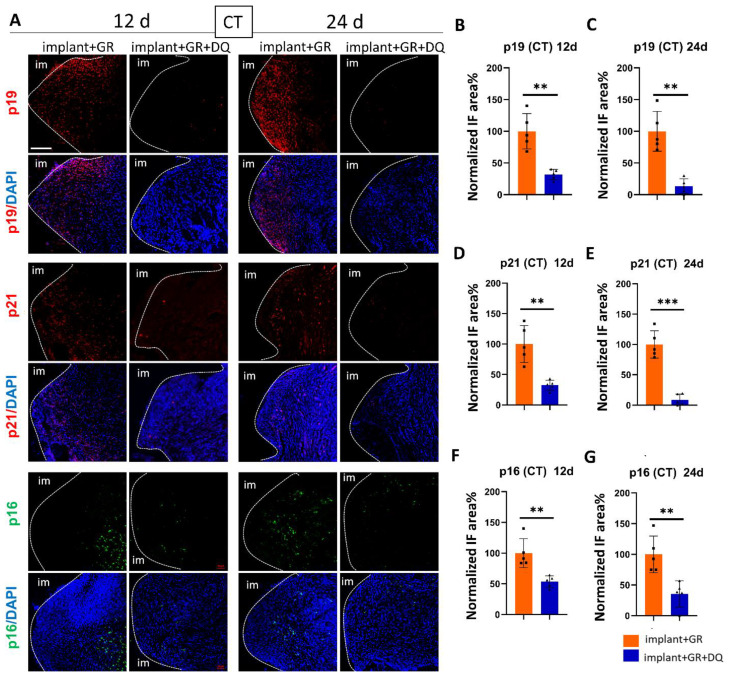
Verification of cellular senescence in CT after D + Q administration. (**A**) Representative immunofluorescent images of CT region against senescent markers p19, p21, p16, and DAPI. im: implant. Scale bar: 100 μm. (**B**–**G**) Quantitative analysis of p19-, p21-, and p16-positive areas in the CT region on day 12 (12 d) and day 24 (24 d) after implantation. Data are presented as mean with SD Student’s *t*-test; ** *p* < 0.01, *** *p* < 0.001. Square and triangle plots are individual value of each group.

**Figure 9:**
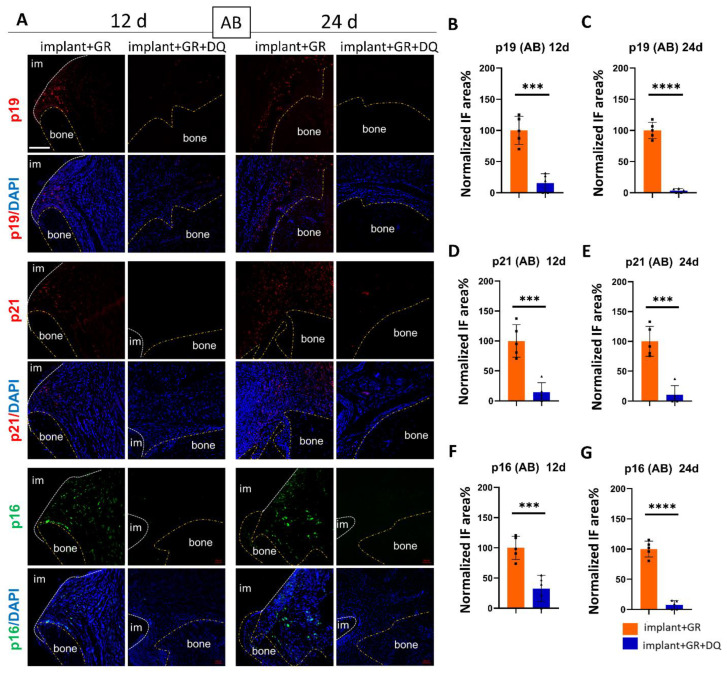
Verification of cellular senescence in AB after D + Q administration. (**A**) Representative immunofluorescent images of the AB region against senescent markers p19, p21, p16, and DAPI. im: implant; bone: alveolar bone. Scale bar: 100 μm. (**B**–**G**) Quantitative analysis of p19-, p21-, and p16-positive areas in the AB region on day 12 (12 d) and day 24 (24 d) after implantation. Data are presented as mean with SD Student’s *t*-test; *** *p* < 0.001, **** *p* < 0.0001. Square and triangle plots are individual value of each group.

We examined whether senolytics could remove senescent cells in peri-implantitis. In the CT region, the p19, p21, and p16 staining areas in the D + Q group were significantly lower than those in the GR group at both 12 and 24 days (Figure 8). In the AB region, as in the CT region, the p19, p21, and p16 staining areas of the D + Q group were significantly lower than those of the GR group at both 12 and 24 days, and the change was more pronounced in the AB tissue than in the CT (Figure 9). These results indicate that D + Q administration reduced the number of senescent cells.

#### 2.4.3. Osteoclast Location by Tartrate-Resistant Acid Phosphatase Staining

**Figure 10:**
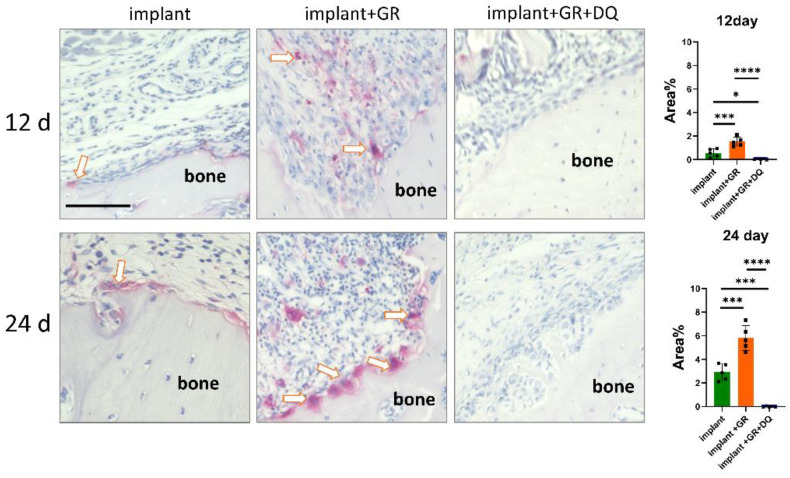
Detection of osteoclasts near AB. Representative tartrate-resistant acid phosphatase (TRAP) staining images of AB and those quantitative data. Arrows: osteoclasts. Scale bar: 100 μm. Quantitative data are presented as mean with SD Student’s *t*-test; * *p* < 0.05, *** *p* < 0.001, **** *p* < 0.0001. Square, triangle and round plots are individual value of each group.

Tartrate-resistant acid phosphatase (TRAP) staining was performed to further analyze the behavior of osteoclasts associated with bone resorption. The TRAP staining positive area was significantly attenuated in the D + Q group compared to the GR group (Figure 10). A few numbers of osteoclasts could be observed after D + Q treatment. These results suggest that the number of osteoclasts decreased by D + Q treatment.

### 2.5. Implant Mobility and Detachment Rate

**Figure 11:**
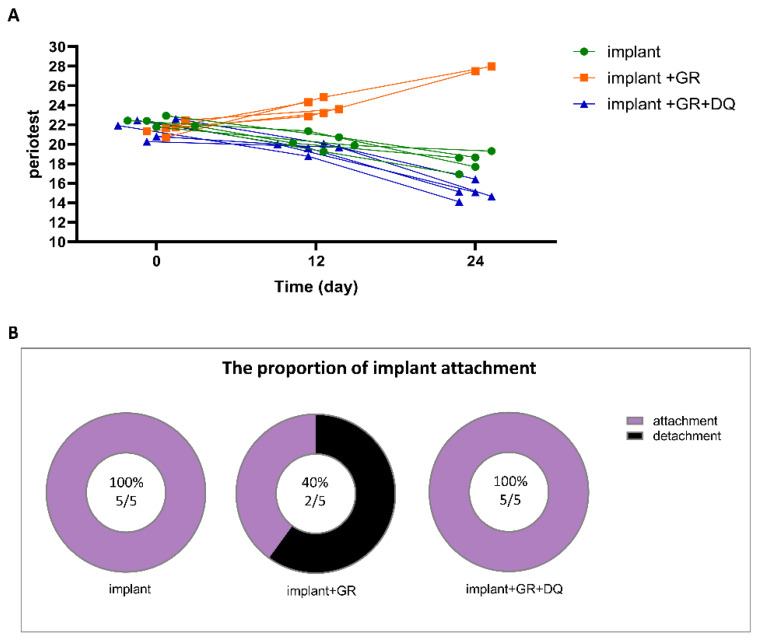
Implant mobility and detachment. (**A**) Implant mobility analysis after implantation (n = 5). (**B**) Implant detachment analysis on day 24 after implantation with implant group (n = 5).

To determine whether the elimination of senescent cells influenced peri-implant upset and dropout, Periotest evaluations were performed (Figure 11). Implant mobility gradually decreased in the implant group and D + Q group but increased in the GR group (Table S2). Intriguingly, although 60% of the implants in the GR group showed detachment by day 24, no implant detachment was observed in the D + Q group (Tables S1 and S3).

## 3. Discussion

The relationship between various inflammatory diseases and senescent cells has been demonstrated in previous studies [26,27]. However, there are few reports on the involvement of senescent cells in peri-implantitis. In this study, the developed peri-implantitis model induced senescent cells. The senolytics removed the senescent cells, decreasing implant mobility and detachment.

Various rat peri-implantitis models have been constructed to control bacteria or plaque adhesion [28,29]. One bacterial approach used *Actinobacillus actinomycetemcomitans* on an alumina-sandblasted implant surface [30]. However, the use of a single bacterium could not mimic the complex bacterial factors caused by plaque. Meanwhile, by promoting plaque adhesion to the implant surface, the ligature wire method could mimic sustained inflammatory cell infiltration and periodontal tissue destruction [29] but failed to evaluate plaque adhesion quantitatively. Consequently, we developed a reproducible rat model of peri-implantitis by combining the plaque evaluation index and GR. The peri-implantitis model showed bone resorption and a two- to three-fold increase in inflammatory cytokines TNF-αand IL-6.

To date, peri-implantitis has been analyzed mainly by examining the downstream signaling network associated with bacterial infection. Inflammatory cytokines such as TNF secreted by bacterial infection promote RANKL, resulting in bone resorption through osteoclast activation [31]. By contrast, few studies have analyzed the relationship between peri-implantitis and senescent cells. In this study, p19-, 21-, and 16-positive senescent cells were observed in the rat peri-implantitis model about three times more than in control. Senescent cells were present in the CT and AB regions. Senescent cells were localized in the submucosal tissue rather than in bone. Thus, we elucidated the presence and localization of senescent cells in peri-implantitis.

Bacteria such as *P. gingivalis* invade through the epithelial barrier disrupted by inflammation, resulting in the secretion of IL-6 and TNF by macrophages and dendritic cells in response to LPS and other components [32]. In peri-implantitis, TNF-α, IL-1α, and IL-6 are significantly upregulated [33]. In this study, D + Q did not remarkably reduce plaque adhesion. D + Q reduced the mean expression of IL-6 and TNF-α but not significantly. However, D + Q administration improved the dysfunction of peri-implantitis. These results suggest that other substances, rather than Il-6 and TNF-α, secreted by senescent cells play a central role in the deleterious function inducing bone resorption in peri-implantitis; DQ is likely to prevent the secretion of such cytokines.

Although we demonstrated the presence of senescent cells and the effectiveness of senolytics in peri-implantitis, further detailed examination is required before the clinical application of senolytics. For example, our study involved a trial using rats, which are different from humans. Cautious verification is necessary for transferring our results to humans. Moreover, the optimal dose and timing of the senolytic administration to avoid side effects in humans are completely unclear. Furthermore, we failed to investigate the type of cells undergoing senescence and the triggers for inducing senescence under peri-implantitis. However, our data should offer a new way to treat peri-implantitis by targeting senescent cells using senolytics. This approach may lead to a better understanding of cellular senescence in peri-implantitis, which can contribute to safe and effective treatment using mini-implants in orthodontics.

## 4. Materials and Methods

### 4.1. Animal Experiments

Eight-week-old male Sprague Dawley rats from SHIMIZU Laboratory (Kyoto, Japan) were used. The animals were housed in environmentally controlled rooms. All animal experiments were conducted with the approval of the Osaka Dental University Institutional Animal Care and Use Committee (Approval No.: 22-02027).

### 4.2. Construction of Rat Peri-Implantitis Model

Under deep anesthesia, a mini-implant (C131205 Characteristics of AbsoAnchor III, Shofu, Kyoto, Japan) with thread length of 5 mm and a diameter of 1.3 mm was placed in the AB 4 mm to the right buccal side of the cervical region of the maxillary anterior tooth (Figure 1A). Each mini-implant penetrated the soft tissue and was implanted into the alveolar bone (Figure 1B). One mini-implant per rats was implanted in each group of 5 rats. A professional new long-hand screwdriver was used for implantation. To develop the peri-implantitis model, the GR was placed between the implant and oral mucosa to allow plaque retention and quantitative evaluation (Figure 1C). The oral cavity was stained for dental plaque (plaque-check gel BR, GC Corporation, Tokyo, Japan) every 4 days, and the dental plaque index was measured.

### 4.3. Sample Collection

At 12 or 24 days after implantation, the tissues were extracted. The extracted samples were fixed in 10% formalin (FUJIFILM Wako Pure Chemical Corporation, Osaka, Japan) and decalcified in EDTA decalcification solution (FUJIFILM Wako Pure Chemical Corporation) at 4 °C.

### 4.4. Histological Staining

All samples were decalcified using Decalcifying Solution B (Cat. No.: LEP2494; FUJIFILM Wako Pure Chemical Co., Osaka, Japan) and then embedded in paraffin. Paraffin sections of 4-µm thickness were obtained by sectioning with KAC Co., Ltd. (Kyoto, Japan) and then subjected to HE and TRAP staining. Staining was performed according to standard protocols at KAC Co., Ltd. HE and TRAP slides were examined with a BZ-9000 digital microscope (Keyence Corporation, Osaka, Japan).

### 4.5. Immunofluorescence

The decalcified samples were embedded and sliced into 7-μm frozen sections using a cryostat (Leica, Wetzlar, Germany) by Kawamoto’s method [34]. The frozen sections were treated by 5% goat serum and 0.1% Triton X-100 in PBS for blocking and permeabilizing

In the analysis of inflammatory cytokines, the primary antibodies of IL-6 (Cat. No.: bs-0782R; Bioss Antibodies Inc., Woburn, MA, USA) and TNF alpha (Cat. No.: 60291-1-lg; Proteintech Group, Inc., Rosemont, IL, USA) were applied to sections overnight at 4 °C. Then, the secondary antibody of ALEXA FLUOR^®^ 594 Conjugated (Goat Anti-Rabbit IgG H&L, Cat. No.: ab150080, Abcam Co., Cambridge, MA, USA) and ALEXA FLUOR^®^ 488 Conjugated (Goat Anti-Mouse IgG H&L, Cat. No.: ab150117, Abcam Co.) was applied to the sections for 1 h at room temperature in the dark. The secondary antibody solutions were decanted and washed three times with PBS for 5 min each in the dark and then stained with DAPI-Fluoromount-G^®^ (No. 0100-20, Southern Biotechnology Associates, Inc., Birmingham, AL, USA).

In the analysis of senescence cell markers, sections were mounted with primary antibody (Anti-CDKN2A/p19ARF Polyclonal Antibody, ALEXA FLUOR^®^ 555 Conjugated (Cat. No.: bs-1174R-A555, Bioss Antibodies Inc.), Anti-P21 Polyclonal Antibody, ALEXA FLUOR^®^ 555 Conjugated (Cat. No.: bs-10129R-A555, Bioss Antibodies Inc.), Anti- CDKN2A/p16-INK4a Polyclonal Antibody ALEXA FLUOR^®^ 488 Conjugated (Cat. No.: bs-23797R-A488, Bioss Antibodies Inc.)) at 4 °C overnight, washed well with PBS, and then mounted with DAPI-Fluoromount-G^®^ (No. 0100-20, Southern Biotechnology Associates, Inc.).

Images were obtained by laser confocal microscopy (LSM-700, Zeiss Microscopy, Jena, Germany). Images were analyzed with ImageJ to assess the ratio of the positive staining area for IL-6, TNF-α, p19, p21, and p16 to the specified measurement area ((antibody positive staining area/measurement area) × 100).

### 4.6. Preparation and Administration of Senolytics Using Dasatinib and Quercetin

Dasatinib (Cat. No.: 11498, Cayman Chemical Co., Ann Arbor, MI, USA) and Quercetin (Cat. No.: sc-206089A, SCB Santa Cruz Biotechnology Inc., Dallas, TX, USA) were mixed with PEG-200 (PEG-200, Cat. No.: ESL3444, FUJIFILM Wako Pure Chemical Co., Osaka, Japan) in Milli-Q with Voltex. The senolytics were orally administered to each rat at a dose rate of 0.25 mL/4 days [35].

### 4.7. Implant Mobility and Detachment

To measure the degree of implant mobility, Periotest M (Medizintechnik Gulden, Modautal, Germany) was used 12 and 24 days after implantation (Figure 1F). The implants were observed every 4 days to determine the implant detachment.

### 4.8. Statistical Analyses

All statistical analyses were performed using GraphPad Prism 8. Further, all results are presented as mean ± standard deviation. To compare four groups, a two-way analysis of variance (ANOVA) was performed. Further, to compare two groups, Student’s *t*-test was performed. The Sidak test was used as a post-hoc test when the ANOVA results were significant.

## 5. Conclusions

Our study demonstrates that the senescent cells appear in peri-implantitis using the rat model with mini-implant and GR. Oral administration of DQ could reduce 70–90% the number of senescent cells (p19, p21, or p16 positive cells) and osteoclasts around the implants, resulting in decreasing implant mobility and detachment even in the peri-implantitis model. The mean of inflammatory cytokines IL-6 and TNF-α decreased but not significantly, suggesting that other SASPs may be associated with the restoration of the stability on the implants. Although we could not elucidate the detailed mechanisms, this study should provide a perspective on the treatment of peri-implantitis and may lead to the development of new methods of prevention and treatment.

## Figures and Tables

**Table 1:** Plaque Scoring Criteria.

Score	Criteria
0	No plaque
1	Flecks of plaque on the edge of the measurement range
2	Definitive line of plaque on the edge of the measurement range
3	A band of plaque covering less than one-third of the measurement range
4	Plaque covering at least one-third but less than two-thirds of the measurement range
5	Plaque covering two-thirds or more of the measurement range

## Data Availability

Not applicable.

## Supplementary Materials

The supporting information can be downloaded at: https://www.mdpi.com/article/10.3390/ijms24032507/s1.
